# Activated Carbon Fiber Incorporated with Metal Ions: Characterization and Biological Interactions In Vitro

**DOI:** 10.3390/ijms27094118

**Published:** 2026-05-05

**Authors:** Letícia Cavassini Torquato, Luiz Augusto Rodrigues dos Santos, Nátaly Domingues Almeida, Clarissa Carvalho Martins Maciel, Glenda Biasotto, Camilla Magnoni Moretto Nunes, Luana Marotta Reis de Vasconcellos, Jossano Saldanha Marcuzzo, Eduardo José de Arruda, Andréa Carvalho De Marco

**Affiliations:** 1 Department of Diagnosis and Surgery, Institute of Science and Technology, São Paulo State University (UNESP), Sao Jose dos Campos 12245-000, SP, Brazil; leticia.torquato@unesp.br (L.C.T.); luiz.a.santos@unesp.br (L.A.R.d.S.); nataly.d.almeida@unesp.br (N.D.A.); camilla.moretto@unesp.br (C.M.M.N.); andrea.marco@unesp.br (A.C.D.M.); 2 Faculty of Exact Sciences and Tecghnology, UFGD—Federal University of Grande Dourados, Dourados 79825-070, MS, Brazil; glendabiasotto@uol.com.br (G.B.); eduardoarruda@ufgd.edu.br (E.J.d.A.); 3 Division of Histology, Institute of Science and Technology, São Paulo State University (UNESP), Campus Sao Jose dos Campos 12245-000, SP, Brazil; luana.marotta@unesp.br; 4 JMHP—Materials Consulting, São Jose dos Campos 12301-600, SP, Brazil; jossano@gmail.com

**Keywords:** carbon fiber, cell viability, osteogenesis, tissue engineering

## Abstract

Due to the constant need to develop biocompatible materials with osteoconductive and osteoinductive properties, the main objective of this study was to evaluate and characterize the carbon fiber obtained from fiber polyacrylonitrile textile carbon fiber (PAN), in the different forms: non-activated carbon fiber felt (NACFF) and activated carbon fiber felt (ACF) with silver (Ag-ACF), gold (Au-ACF), copper (Cu-ACF), palladium (Pd-ACF) and platinum (Pt-ACF), on the cell behavior and osteogenesis of mesenchymal cells. For characterization: scanning electron microscopy (SEM), energy dispersive spectroscopy (EDS) and Raman analysis. In vitro analysis was performed on rat mesenchymal stem cells. For each experimental group, 5 wells (*n* = 5) were made where cell proliferation (CP): cell viability (CV), mineralization nodule formation (MNF), total protein content (PT) and alkaline phosphatase activity (APC) were quantified, and cell morphology was analyzed by direct fluorescence, genotoxicity and cell interaction by SEM. The data passed the normality test and was followed by the one-way ANOVA test, followed by the Tukey test, using the conventional significance level of 5%. All the samples were statistically similar in terms of cell proliferation, except for the Ag-ACF group in relation to the control group (C). For cell viability, C obtained greater viability than the other groups, while ACF obtained a statistical difference and was superior to the Ag-ACF, Cu-ACF, Pt-ACF groups, being statistically similar to the Au-ACF and Pd-ACF groups. In the evaluation of ACP, the Ag-ACF and Cu-ACF groups were lower than the C, and other groups; for the characterization tests Au-ACF and Pd-ACF showed a more homogeneous metal distribution compared to the other groups. Cu-ACF and Ag-ACF showed some toxicity and low induction of osteoblastic differentiation. Although platinum showed relative cellular viability, a high micronucleus count was reported for this ion. In conclusion, ACF has the potential to be developed as a future biomaterial with good cell viability. Carbon fibers incorporated with gold and palladium ions showed potential for future application as supports for bone repair.

## 1. Introduction

The number and complexity of biomaterials have been increasing annually, and their development is crucial for advancements in healthcare [1]. Biomaterials used for bone repair typically possess osteoconductive properties, serving as biocompatible scaffolds that support cell migration, proliferation, and differentiation for tissue regeneration [2,3]. With a focus on synthetic biological materials as alternatives to autogenous grafts, carbon fibers have emerged as promising biomaterials due to their mechanical properties, lightness, strength, flexibility, and compatibility with other materials [4].

In the late 1970s, carbon fibers were introduced to biomaterials studies due to their lightness, high strength, flexibility, and compatibility with other biomaterials. Carbon fibers offer benefits such as varied presentations, high radiolucency, good biocompatibility, and long stability in vivo [5].

Carbon fibers are non-woven fabrics made from a textile precursor polyacrylonitrile textile carbon fiber (PAN) in a 3D framework as a sustainable carbon polymer (graphite). They are versatile and can meet various technological needs due to their density, resistance, stability, and biocompatibility. Carbon fibers have properties suitable for numerous applications, especially in bone regeneration scaffolds [6]. Zhang et al., 2023 [7] showed that carbon fiber alone, without the incorporation of metals, presented hemocompatible, biocompatible, and supported cell attachment, being considered for use as a scaffold in bone regeneration [8].

In addition to the use of carbon, there are promising studies on desirable properties of biomaterials impregnated with metal ions, the method of impregnation is considered a simple, economical and safe way to increase bone regeneration, increase in bone volume and increase in the number of bone trabeculae [8,9]. For example, copper can contribute to cell adhesion, proliferation, migration and cell differentiation of mesenchymal stem cells into osteogenic cells, along with its role in stimulating collagen fiber deposition and angiogenesis, have made it a focal point of interest in the field of bone tissue engineering; in addition to antibacterial and anti-infectious properties similar to silver, which is also able to accelerate the process of osteogenesis [8,10]. Metal ions, such as copper, silver and gold, in biomaterials have shown positive results in bone repair because they have the potential to promote the induction of osteoblastic cell differentiation [11], an increased number of blood vessels and antibacterial action [12,13]. In an in vivo study, gold exhibits bone stimulation characteristics by increasing osteoblast production and bone mineral density (BMD) [14,15,16].

The possibility of anticancer properties of platinum and palladium ions has been studied and shown promising results [15,16]. The scaffolds with Pd nanoparticles provided the differentiated grown osteoblast cells with enough mechanical support and stability and the cells had a regular form and were highly dense [17]. However, there is still no full knowledge of the specific mechanisms of the effectiveness of the impregnation of these ions in bone regeneration [18].

Therefore, the present study aims to evaluate activated carbon fiber felt impregnated with different metal ions in differentiation with mesenchymal cells in vitro, with the intention of assisting in the development of a new biomaterial non-woven with adequate biological properties for bone repair.

## 2. Results

### 2.1. Scanning Electron Microscopy (SEM-EDS)

**Figure 1:**
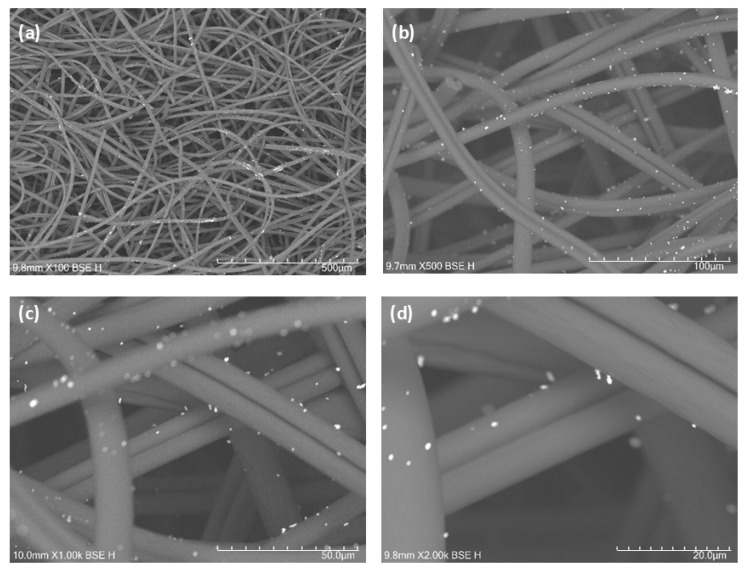
(**a**–**d**) SEM images of activated carbon fibers (ACF) decorated with gold nanoparticles (AuNPs) at different magnifications, with the distribution of AuNPs on the fiber structure.

**Figure 2:**
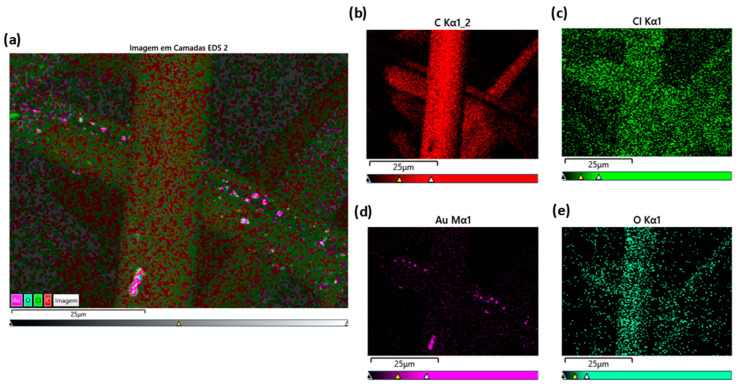
EDS mappings (**a**) layered image of Au-ACF, chemical elements (**b**) carbon, (**c**) chlorine; (**d**) gold; (**e**) oxygen.

**Figure 3:**
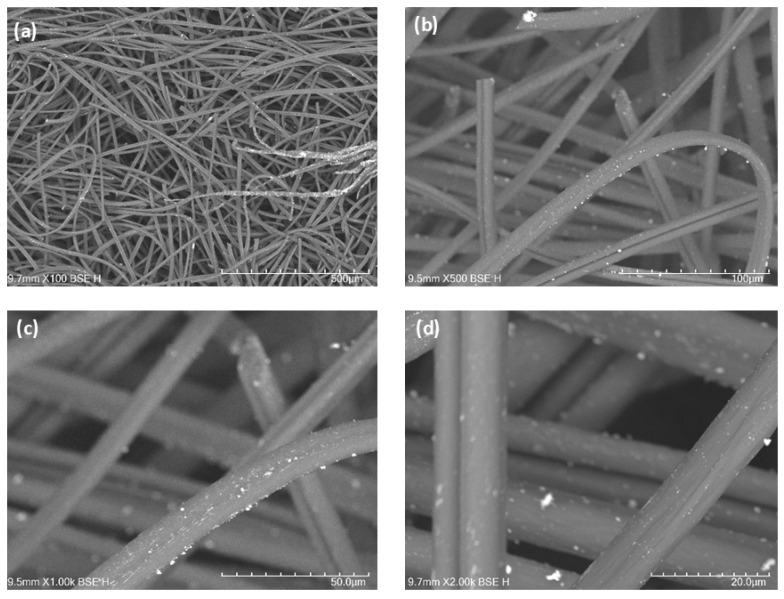
(**a**–**d**) SEM images of activated carbon fibers (ACF) decorated with palladium nanoparticles (PdNPs) at different magnifications, with the distribution of PdNPs on the fiber structure.

**Figure 4:**
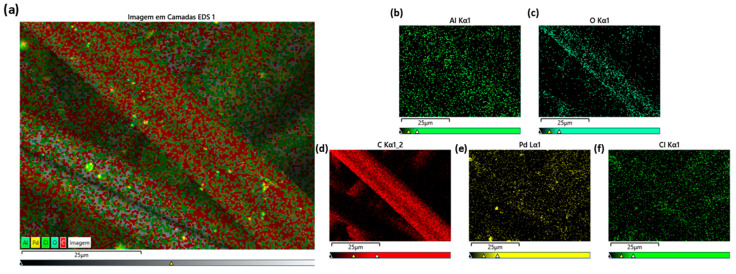
EDS mappings (**a**) layered image of Pd-ACF; chemical elements (**b**) aluminum; (**c**) oxygen; (**d**) carbon; (**e**) palladium; (**f**) chloride.

Scanning electron microscopy and EDS analysis revealed a homogeneous distribution of gold (Au) nanoparticles (Figure 1 and Figure 2), palladium (Pd) nanoparticles (Figure 3 and Figure 4). Besides their uniform distribution, the metallic particles also exhibit a standardized size and are well-dispersed across various regions of the fibers.

The EDS spectra obtained from carbon fibers with Au and Pd are illustrated in the figures below. Figure 2 shows the elements detected in the sample with Au: carbon, oxygen, chlorine, and gold.

### 2.2. Raman Analysis

**Figure 5:**
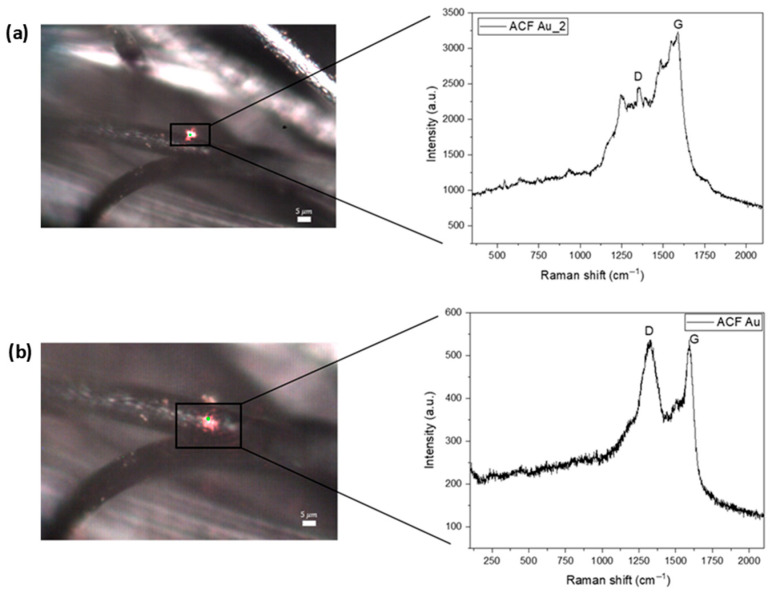
Raman spectra of the Au-ACF group. (**a**) Image focused on the Au nanoparticle at carbon fiber and Raman spectrum collected from a single Au nanoparticle, highlighting the localized interaction and signal enhancement due to the presence of Au. (**b**) General image of carbon fiber with Au nanoparticle and the general Raman spectrum of the ACFs, showing the characteristic D and G bands of the carbon structure.

**Figure 6:**
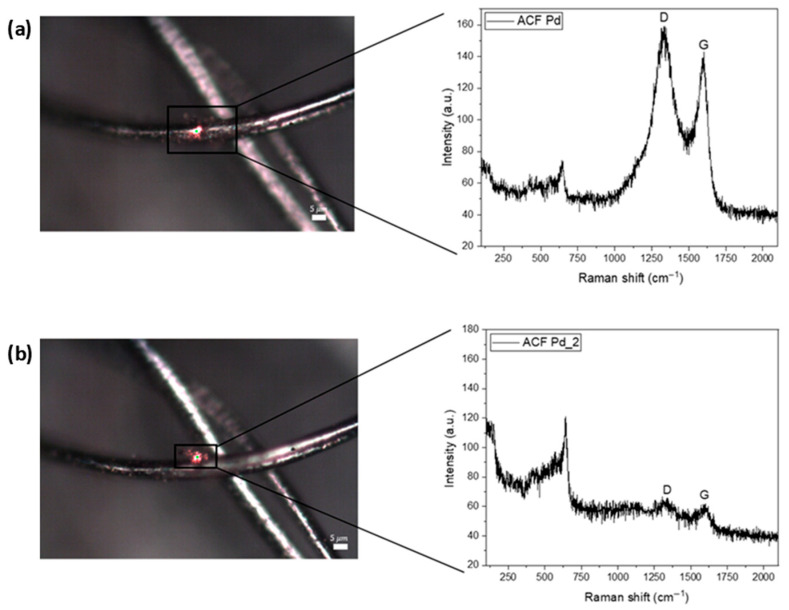
Raman spectra of the Pd-ACF group. (**a**) General image of carbon fiber with Pd nanoparticle and general Raman spectrum of the ACFs showing the characteristic D and G bands of the carbon structure. (**b**) Image focused on the Pd nanoparticle at carbon fiber and Raman spectrum collected from a single Pd nanoparticle, highlighting the localized interaction and signal enhancement due to the presence of Pd.

The figure below shows the Raman spectrum of the carbon fiber activated with gold (Au) (Figure 5) and palladium (Pd) nanoparticles (Figure 6). The laser power used was 0.45 mW and the number of accumulations was 3 × 120 s. The G peak corresponds to the 1st order Raman, with the frequency of the G band (~1586 cm^−1^), and occurs due to the stretching of the CC bond of all pairs of sp^2^ atoms. The spectra also show the D band (~1348 cm^−1^), characterized by a disturbance that is activated by defects. At specific points on the fibers, the characteristic bands of each metal can be detected.

### 2.3. Cell Proliferation

**Figure 7:**
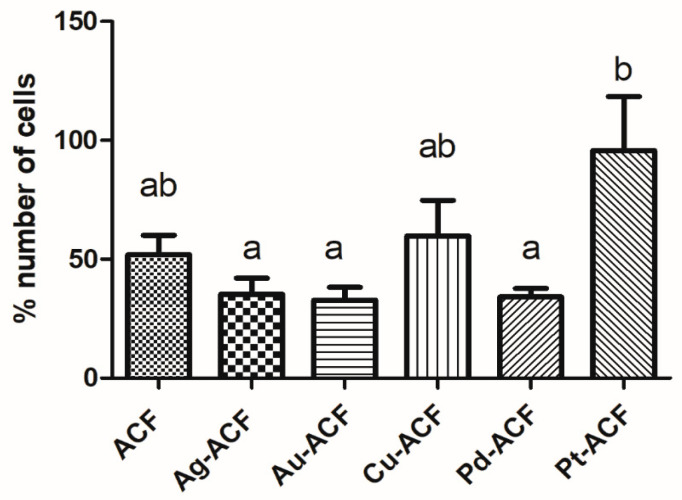
Graph with mean and standard deviation of the proportion of number of cells per group in relation to the control group. Significant statistical difference is represented as different lowercase letters, one-way ANOVA, *p* < 0.05.

In the cell proliferation test, most groups demonstrated similarity in the results in relation to the proportion of the number of cells in the control group. Only the Pt-ACF group presented a statistically significant difference in relation to the Ag-ACF group (*p* < 0.05) with higher results (Figure 7).

### 2.4. Cellular Interaction

**Figure 8:**
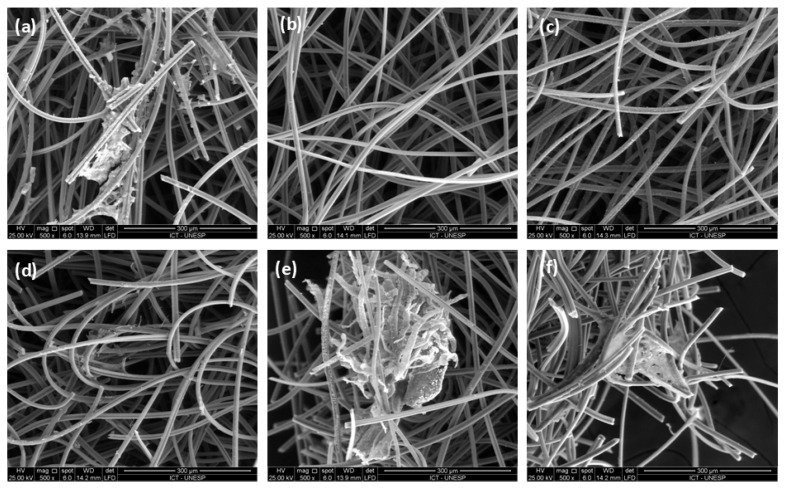
SE Scanning Electron Microscopy, 500× magnification. Adherent and scattered cells can be observed in almost all samples. (**a**): NACFF, (**b**): Ag-ACF, (**c**): Au-ACF, (**d**): Cu-ACF, (**e**): Pd-ACF, (**f**): Pt-ACF.

In scanning electron microscopy, the cells, when visible, showed characteristics of adherence to the biomaterial and cellular spread, often connecting the fibers of the felt. It was possible to observe adhered and spread cells in almost all samples; only the Ag-ACF and Au-ACF groups did not present adhered cells (Figure 8).

### 2.5. Cell Viability

**Figure 9:**
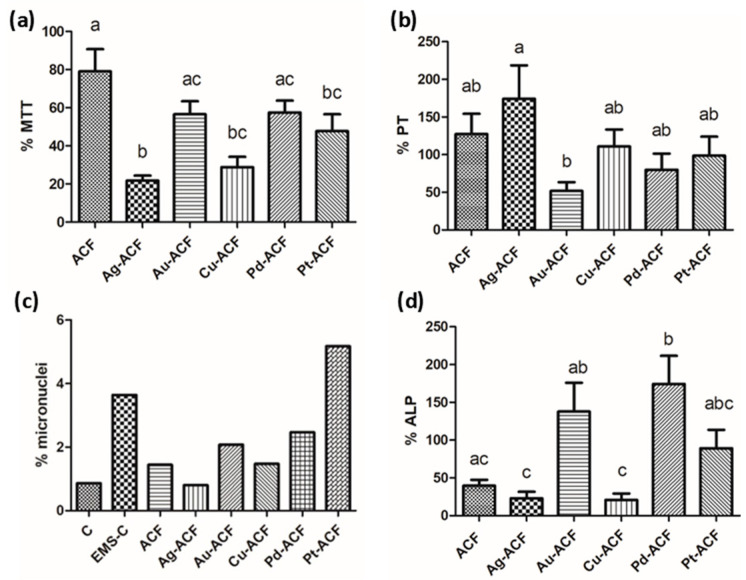
(**a**) Graph with mean and standard deviation of the proportion of cell viability (MTT) in relation to the control group. (**b**) Graph with mean and standard deviation of the proportion of total protein (PT) content in relation to the control group. (**c**) Graph representing the proportion of micronuclei counts per group. (**d**) Graph with mean and standard deviation of the proportion of alkaline phosphatase activity (ALP) in relation to the control group. Significant statistical difference is represented as different lowercase letters, one-way ANOVA, *p* < 0.05.

Among the proposed biomaterials, the ACF group showed a statistically significant difference compared to the Ag-ACF, Cu-ACF, and Pt-ACF groups (*p* < 0.05), but was statistically similar to the Au-ACF and Pd-ACF groups, representing the highest rate of cell viability (Figure 9a). The Ag-ACF group was statistically similar to Cu-ACF and Pt-ACF (*p* > 0.05), showing the lowest cell viability proportion among the biomaterials.

### 2.6. Total Protein Content

In the evaluation of total protein content, a statistical difference was found only between the Ag-ACF and Au-ACF groups (*p* < 0.05), with the gold ion group showing lower total protein production expression (Figure 9b).

### 2.7. Alkaline Phosphatase Activity

In the test to evaluate the activity of alkaline phosphatase, the Au-ACF, Pd-ACF, and Pt-ACF groups were statistically similar to the ACFF group (*p* < 0.05) but showed higher proportions of alkaline phosphatase activity compared to the control group (Figure 9d).

### 2.8. Genotoxicity

The micronuclei were counted by group, and the sample that presented the highest proportion of micronuclei was that of palladium ions (Figure 9c), exceeding the count of the EMS control. The rest of the groups presented a lower number of micronuclei.

### 2.9. Formation and Quantification of Mineralization Nodules

**Figure 10:**
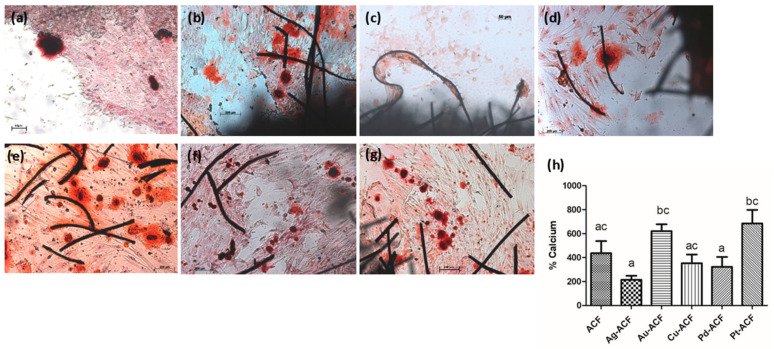
Representative photomicrographs of mineralization nodules impregnated with Alizarin red dye (×10). (**a**): Cellular control; (**b**): NACFF; (**c**): Ag-ACF; (**d**): Au-ACF; (**e**): Cu-ACF; (**f**): Pd-ACF; (**g**): Pt-ACF. (**h**) Graph with mean and standard deviation of the quantification of mineralization nodules in relation to the control group. Significant statistical difference is represented as different lowercase letters, one-way ANOVA, *p* < 0.05. Scale bar = 200 μm.

All groups presented mineralization nodules, which in the samples were concentrated near the felt fibers (Figure 10). The Ag-ACF and Pd-ACF groups showed lower calcium quantification than the Au-ACF and Pt-ACF groups (*p* < 0.05).

## 3. Discussion

The main objective of this study was to evaluate the different presentations of activated carbon fiber felt obtained from textile PAN fiber in its different forms, in the osteogenesis of mesenchymal cells in order to obtain new information for the development of a new biomaterial by tissue engineering, with adequate biological properties.

In view of the pioneering nature of this project, due to the use of activated carbon fiber felt (ACFF) obtained from textile PAN fiber as a possible biomaterial, it was challenging to find similar in vitro studies in the literature, especially those that evaluated the material in the different presentations with the incorporation of metal ions; Therefore, in order to better understand the results obtained in the tests, it was possible to correlate the results obtained with other studies made with carbon-based materials of other modalities and with studies that evaluated the properties of the same metals of the ions used in this project.

The application of carbon-based materials in the creation of new biomaterials was shown in an in vitro study by Crisan et al. [19], where biocompatibility of graphene composite with gold and hydroxyapatite nanoparticles in osteoblasts was observed, and its low cytotoxicity, similar to what was observed in the present study, in which carbon fiber felts showed good cell viability, and the only felts that showed low viability were incorporated with silver and copper, and this result may be more associated with the ions than with the fiber itself.

In terms of fiber characterization, the SEM and EDS tests showed that the Au-ACF and Pd-ACF groups had homogeneous dispersion in terms of the size and distribution of the metal particles over the entire surface of the fiber, compared to the Ag-ACF, Cu-ACF and Pt-ACF groups, which were only dispersed at certain points on the surface. This may have influenced the performance of these groups (Ag-ACF, Cu-ACF, Pt-ACF), since the deposition of these metals may have been heterogeneous and the particles may also have been irregular in size [20].

In addition to the low cytotoxicity of carbon-based materials, in a study by Aoki et al. [21], using a thin web of carbon fiber as a scaffold for bone regeneration, it was found that this carbon-based material, of presentation and structure similar to the felts of this study, by scanning electron microscopy images was demonstrated to be an efficient scaffold for repair of bone defects in iliac bone of rats, being a strong candidate for use in bone repair therapy. Therefore, by the structural similarity of the felt used in this study with the carbon fiber web of the study by Aoki et al. [21], it is possible to infer, together with the in vitro results of low cytotoxicity of the textile PAN fiber felt (ACFF), a high potential of the samples of this study for application in bone repair therapy.

According to Junior et al., 2017 [22], the activation process, used in the preparation of felts, forms grooves in the material increasing the exposed surface and the number of pores and their distribution, and this porosity is an aspect of interest when it comes to the development of 3D scaffolds, because it allows cell adhesion, proliferation and osteogenic differentiation [22,23]. Previous work from our group with PAN-based activated carbon fibers under similar activation conditions has reported significant porosity and surface area values, confirming that the activation process generates a porous structure suitable for biological applications [22,24]. In the scanning electron microscopy images, it was possible to observe the cellular adhesion to the material in all groups of samples; besides that, the cell proliferation test showed a statistically significant difference only between the control group and the carbon fiber felt activated with silver ions.

Regarding the incorporation of metal ions, it was possible to see in this study that the felt incorporated with gold ions showed good viability and indication of induction to osteoblastic cell differentiation, inferred by the results of the nodule quantification tests and the alkaline phosphatase activity. The property of induction of cell differentiation in osteoblasts of gold ions was also verified in the in vitro study by Crisan et al. [19], where the incorporation of gold into carbon-based materials was favorable for the induction of osteoblastic differentiation, which demonstrates a positive trend towards the application of these ions in bone tissue engineering.

Regarding silver, copper and palladium ions, in a study by Milheiro et al. [25] on the cytotoxicity of several ions in mouse fibroblast cells through the MTT assay, it was shown that palladium ions have good viability up to a concentration of 100 ppm while silver and copper ions, demonstrated cytotoxicity from low concentrations such as 10 ppm, and these results are compatible with those presented in the present study.

The palladium group showed good cell viability, in addition to low total protein production, high rate of alkaline phosphatase activity and reasonable quantification of mineralization nodules, similar to the gold group, and the copper and silver ion groups showed high total protein content, low cell viability and low rate of alkaline phosphatase activity and quantification of mineralization nodules. These results of the copper and silver groups may be associated with the production of apoptotic proteins that were accounted for by the total protein test, and the low viability, low alkaline phosphatase activity and low quantification of nodules, may be associated with certain cytotoxicity of the ions and low induction of osteoblastic differentiation.

Regarding these aspects, both silver (Ag^+^) and copper (Cu^2+^/Cu^+^) ions are transition metals capable of inducing oxidative stress through the generation of reactive oxygen species (ROS). These ions undergo redox cycling reactions that lead to the formation of free radicals, resulting in oxidative damage to lipids, proteins, and nucleic acids. The increased ROS levels trigger a cascade of cellular events, including mitochondrial dysfunction and activation of apoptotic pathways [26,27,28,29]. Studies employing silver nitrate (AgNO_3_) have demonstrated inhibition of DNA synthesis and a decrease in cellular proliferative capacity. Furthermore, the “Trojan horse” mechanism has been frequently described for silver nanoparticles (AgNPs), which release Ag^+^ ions that interact with plasma membrane components, increasing membrane permeability and facilitating intracellular entry. This process leads to loss of membrane integrity, accumulation of oxidative lesions, and induction of apoptosis [28,29]. These observations may provide a mechanistic rationale and further substantiate the findings of the present study with respect to cell viability.

The results presented by the genotoxicity test should be read with some caution, due to the toxicity of silver and copper ions, the number of cells was reduced at the time of accounting for micronuclei, which may have generated some bias in the results presented, since the sample impregnated with platinum presented relatively good cell viability when compared to other fibers.

The elevated frequency of micronuclei in the Pt-ACF group is likely due to the ability of platinum to bind to DNA and interfere with the progression of mitosis. In stem cells, however, this phenomenon should not be interpreted exclusively as an indicator of cell death, but also as a marker of genotoxic stress and the activation of repair pathways. Therefore, increased micronucleus formation may reflect complex genotoxic interactions that extend beyond direct cytotoxic effects. In addition to its direct interaction with DNA, platinum in metallic or nanoparticulate form can promote the generation of reactive oxygen species (ROS), thereby contributing to oxidative stress and indirect damage to the cellular genome [30,31].

Given that the toxic potency of metal ions is strongly influenced by several factors, including concentration, exposure time, chemical form (free ion versus nanoparticle), medium composition, and cell type, future research should encompass additional genotoxicity assays to further validate the findings of the present study. In this context, complementary characterization methods capable of accurately assessing porosity would also be of great relevance. Furthermore, the development and optimization of strategies for incorporating metal ions into activated carbon fibers represent an important avenue for investigation.

Activated carbon fiber incorporated with metal ions has attracted increasing attention in biomedical applications, especially in the field of bone regeneration. Its biocompatibility and favorable mechanical properties may give it potential for use as a bone filler, promoting structural support and facilitating osteoconduction. In addition, when used as a scaffold for bone tissue engineering, activated carbon fiber can act as a three-dimensional matrix capable of supporting the adhesion, proliferation, and differentiation of osteogenic cells. The incorporation of metal ions, such as gold, palladium, and even copper or silver, can enhance its bioactive properties, favoring the formation of a mineralized matrix and, in some cases, conferring antimicrobial activity, which can contribute to a microenvironment more favorable to bone regeneration.

## 4. Materials and Methods

### 4.1. Preparation of Activated Carbon Fiber

PAN fibers of 5.0 dtex in a cable of 200 thousand filaments were thermally oxidized and used as raw material to produce felt. The raw material was placed in a sample holder and inserted into an electric tubular furnace for carbonization (900 °C) for 30 min, and then activated in a CO_2_ atmosphere at 1000 °C for 50 min. At the end, the Activated Carbon Fiber Felt (ACFF) is obtained. The carbon fibers were placed in direct contact with metallic salts for the direct deposition of reduced metals on the surface. ACF samples were immersed in 50 mL of 0.1 M metal salt solutions and kept under static conditions at 25 °C for 48 h to achieve metal impregnation [32]. The samples were thoroughly washed with ultrapure water and dried in an oven at 50 °C for 24 h. They were cut into circles to carry out in vitro experiments. At this point, the felt is ready to go through the adsorption process with ion solutions, and then the following fibers are obtained: Silver-activated carbon fiber felt (Ag-ACF), Gold-activated carbon fiber felt (Au-ACF), Copper-activated carbon fiber felt (Cu-ACF), Palladium-activated carbon fiber felt (Pd-ACF), and Platinum-activated carbon fiber felt (Pt-ACF). From the preparation of the carbon fiber felt, samples of 4 mm in diameter and 2 mm in height were obtained [33].

The samples were separated into groups according to the forms of presentation of the material. They were previously packed and sterilized in an autoclave (121 °C for 20 min) before being sent for in vitro analyses.

### 4.2. Scanning Electron Microscopy (SEM-EDS)

The samples were analyzed using a Hitachi TM4000 Plus scanning electron microscope (Hitachi, Tokyo, Japan). Microscopy images were obtained at magnifications of 100×, 500×, 1000×, and 2000×, initially providing a general view of the fibers and, at higher magnifications, revealing detailed surface features with nanoparticles. The deposition of nanoparticles on the fibers increases their surface area, making these materials highly significant for various applications. Energy Dispersive Spectroscopy (EDS) analysis confirmed the presence of the deposited metals and quantified the elemental composition of each sample.

### 4.3. Raman Analysis

Surface Enhanced Raman Spectroscopy analyses (SERS) were performed using a Horiba HR 800 Evolution micro-Raman—AFM Horiba spectrometer with a 514 nm laser (Horiba, Palaiseau, France), with the laser power values 0.45 mW and the number of accumulations 3 × 120 s. This characterization technique enables the identification of the chemical structure of the analyzed material. The obtained information is extracted from the scattering of electromagnetic radiation after its interaction with the material, which can be either inorganic or organic.

### 4.4. In Vitro Biological Assays

This study was conducted in accordance with the Ethical Principles for Animal Experimentation and approved by the Ethics Committee on Animal Use of the Institute of Science and Technology of São José dos Campos—ICT/UNESP (n° 11/2021). The description of this work was made following the guidelines of Animal research: Reporting in Vivo Experiments—ARRIVE [34].

### 4.5. Cell Culture

The mesenchymal cells were obtained from the femurs of 9 adult male rats (albino variation, Wistar strain) weighing approximately 300 g at around 90 days of age. The rats were housed in appropriate cages and had access to food and water ad libitum until euthanasia The animals were euthanized by administering a triple dose of a solution of xylazine hydrochloride 2% (10 mg/kg Anasedan—Ceva Brazil, Paulínia, Brazil) and ketamine 10% (100 mg/kg Dopalen—Ceva Brazil) intramuscularly. Subsequently, the femurs were removed for cell culture.

The bone marrow cells from the femurs were isolated using minimum essential alpha MEM culture medium (Gibco^®^—Life Technologies, Waltham, MA, USA) supplemented with 10% Fetal Bovine Serum (FBS) (Cultilab Ltda, São Paulo, Brazil) and gentamicin (500 μg/mL) (Gibco^®^—Life Technologies, USA) [35,36].

Before plating, the samples were distributed on the 96-well plate. First passage cells plating was performed with approximately 5000 cells/well in an osteogenic medium (*n* = 5) containing 5 mg/mL of ascorbic acid (Neon, Neon^®^ Comercial, Suzano, Brazil) and 2.16 g of beta glycerophosphate (Sigma-Aldrich^®^ ref 50020, Merck KGaA, Darmstadt, Germany). During cell culture, the cells were incubated at a temperature of 37 °C, with atmospheric humidity containing 5% CO_2_.

### 4.6. Cell Proliferation

Cells were cultured in the plate wells for 24 h. The culture medium was aspirated, and the samples were washed with PBS (Gibco^®^—Life Technologies, Baltimore, MD, USA) three times. After washing, 200 μL of 0.25% trypsin solution (Cultilab Ltda, Campinas, Brazil) was pipetted to detach the cells from the sample, then the plate was placed in a CO_2_ incubator for 15 min. The cells were counted using the Neubauer chamber and Trypan Blue dye, and cells that allowed the dye to be incorporated by the membrane were considered non-viable [37].

### 4.7. Cellular Interaction

After 7 days of culture, the cellular interaction with the biomaterial was evaluated by FE-SEM (Field Emission Scanning Electron Microscopy) (Zeiss—EVO MA10, São Paulo, Brazil). The samples were washed three times with PBS and then chemically fixed with 4% paraformaldehyde at room temperature for 20 min. Subsequently, the samples were dehydrated using an ascending series of ethanol. Prior to analysis, they were coated with a thin layer of gold using a sputter-coating system.

### 4.8. Determination of Cell Viability

After 7 days of cell culture, aliquots of MTT [3-(4,5-dimethylthiazol-2-yl)-2,5-diphenyltetrazolium bromide] (Sigma Aldrich^®^, Merck KGaA, Darmstadt, Germany) at 0.5 mg/mL in culture medium were prepared. The primary cultures were then incubated with this solution for 4 h at 37 °C. Subsequently, the MTT solution was removed, and 500 μL of DMSO solution (Dimethyl Sulfoxide, Gibco—Life Technologies, Baltimore, MD, USA) was added to the wells and left for 10 min in a CO_2_ incubator at 37 °C. The plate was then agitated for 10 min. Aliquots of 100 μL were taken from the wells and transferred to a 96-well plate (Greiner CELLSTAR^®^, Merck KGaA, Darmstadt, Germany) for colorimetric measurement in a microplate reader at a wavelength of 570 nm (Biotek^®^ EL808IU, BioTek Instruments, Inc., Winooski, VT, USA). The data were recorded as absorbance values and stored for statistical analysis.

### 4.9. Total Protein Content

After 10 days of culture [38], the wells were washed three times with PBS, and filled with 2 mL of 0.1% sodium lauryl sulfate (Sigma-Aldrich^®^, Merck KGaA, Darmstadt, Germany). After 30 min, 1 mL of the solution from each well was mixed with 1 mL of Lowry’s solution (Sigma-Aldrich^®^, Merck KGaA, Darmstadt, Germany) and left for 20 min. Then, 1 mL of Folin and Ciocalteau reagent (Sigma-Aldrich^®^, Merck KGaA, Darmstadt, Germany) was added to the mixture for 30 min. Next, absorbance was measured at 680 nm in a spectrophotometer (Micronal AJX 1900, Micronal^®^, S.A., São Paulo, Brazil) and the total protein content was calculated from a standard curve determined by bovine albumin and expressed in μg/mL.

### 4.10. Alkaline Phosphatase Activity

After 10 days of cell culture [39], alkaline phosphatase activity was measured by the release of thymolphthalein through hydrolysis of the thymolphthalein monophosphate substrate. A commercial kit from Labtest Diagnóstica^®^ S.A in Lagoa Santa, Minas Gerais, Brazil was used following the manufacturer’s instructions. Initially, 50 μL of thymolphthalein monophosphate was mixed with 0.5 mL of 0.3 M diethanolamine buffer, pH 10.1. A 50 μL aliquot of the lysates obtained from each well was added to the solution and left for 10 min at 37 °C in a water bath. For color development, 2 mL of calcium carbonate (Na_2_CO_3_) and 0.25 M sodium hydroxide (NaOH) were added. Absorbance was measured in a spectrophotometer (Micronal^®^ AJX 1900, Micronal, S.A., São Paulo, Brazil) using a wavelength of 590 nm, and alkaline phosphatase activity was calculated from a standard curve using thymolphthalein on a scale of 0.012 to 0.4 μmol thymolphthalein/h/μg protein.

### 4.11. Genotoxicity Assessment

The genotoxicity test was performed on the third day of cell culture, in accordance with the OECD Guideline (TG 487), updated in 2016. The cells were plated in 24-wells (2 × 10^5^ cells/well) containing 500 µL of minimal essential medium supplemented with 10% fetal bovine serum, antibiotics, and kept in an incubator at 37 °C with 5% CO_2_. The cells in the positive control group were submitted to a 5 mM ethymethane sulfonate (EMS; Sigma-Aldrich^®^, Darmstadt, Germany) solution, while the negative controls received only the minimal essential medium. In the experimental wells, the cells were treated with cytocalasin B (Sigma-Aldrich^®^) at a concentration of 6 µg/mL. The plates were incubated for 24 h, and the wells were washed with phosphate-buffered saline (PBS), and fixed with methanol and acetic acid solution (3:1) for 10 min. After another wash with PBS, a drop of DAPI (Fluorshield^®^ with DAPI, Sigma-Aldrich) was added. The analysis was performed under a fluorescence microscope (Carl Zeiss Microlimaging GmbH—Axiovert 40C, Jena, Germany), considering 2000 cells per well. The data obtained are presented as a proportion (%) of the number of micronuclei presented in relation to the number of nuclei and cells.

### 4.12. Formation and Quantification of Mineralization Nodules

After 10 days of culture, the adhered cells were fixed in a 10% formaldehyde solution for 2 h. After fixation, the samples were dehydrated with a gradual series of alcohol and stained with 2% Alizarin Red S (Sigma-Aldrich^®^, Merck KGaA, Darmstadt, Germany) at pH 4.2 for 10 min. The plate wells were photographed under an Axio HBO 100 light microscope (Carl Zeiss^®^ Microlimaging GmbH—Axiovert 40C, Germany) at a 200-fold magnification.

The quantification of mineralized formations was performed as follows: 800 μL of 10% acetic acid was added, and the plates were incubated at room temperature with agitation for 30 min. All contents of each well were transferred to centrifuge microtubes with 1.5 mL and vortexed (Vortex QL—901, Biomex Biotecnologia, Ribeirão Preto, SP, Brazil) for 30 s. The microtubes were then placed in a water bath (Dubnoff Metabolic Bath—MA-095/CF, Marconi Equipamentos Para Laboratórios Ltd.a, Piracicaba, SP, Brazil) and heated for 10 min at 85 °C, followed by transfer to ice for 5 min. Subsequently, they were centrifuged (Labnet Centrifuge—HERMLE Z 300K, HERMLE Labortechnik GmbH, Wehingen, Germany) for 20 min, and 100 μL of the supernatants were transferred to a 96-well plate. In each well, 40 μL of 10% ammonium hydroxide was added. The reading was performed in a microplate reader (Biotek—EL808IU, BioTek Instruments, Inc., Winooski, VT, USA) at a wavelength of 405 nm.

### 4.13. Statistical Analysis

The data obtained from the test groups were calculated as proportions in relation to the control group. They were then presented as mean and standard deviation, underwent a Shapiro–Wilk test to verify normality, and were analyzed using a one-way ANOVA test. This was followed by Tukey’s test with a significance level of 5%.

## 5. Conclusions

It is possible to conclude that activated carbon fiber felts have potential for development as a future biomaterial non-woven, with good cell viability. Carbon fibers incorporated with gold (Au-ACF) and palladium ions (Pd-ACF) have demonstrated potential for future application as scaffolds for bone repair.

## Data Availability

The original contributions presented in this study are included in the article. Further inquiries can be directed to the corresponding author.
